# Recurrent flash pulmonary edema in unilateral renal artery stenosis with contralateral kidney shrinkage: A case report

**DOI:** 10.1097/MD.0000000000035228

**Published:** 2023-09-22

**Authors:** Ji Eun Cheon, Hyo Jin Kim

**Affiliations:** a Department of Internal Medicine, Pusan National University Hospital, Busan, Korea; b Department of Internal Medicine, Pusan National University School of Medicine, Yangsan, Korea; c Biomedical Research Institute, Pusan National University Hospital, Busan, Korea.

**Keywords:** flash pulmonary edema, renal artery stenosis, renal artery stenting, single-functioning kidney

## Abstract

**Rationale:**

Flash pulmonary edema is a critical medical condition characterized by sudden and severe fluid accumulation in the lungs, which poses an immediate and life-threatening emergency. This can arise from a variety of underlying causes. This manuscript presents a case of recurrent pulmonary edema that was successfully managed through the insertion of a renal artery stent.

**Patient concerns:**

A 78-year-old woman visited the emergency room with recurrent acute dyspnea. Computed tomography renal angiography revealed renal artery stenosis of a single-functioning kidney.

**Diagnoses:**

Flash pulmonary edema caused by renal artery stenosis of a functioning single kidney.

**Interventions:**

Percutaneous transluminal angioplasty and stenting were performed for the renal artery stenosis.

**Outcomes:**

The patient’s kidney function rapidly improved, and she has been free of flash pulmonary edema for 2 years.

**Lessons:**

Flash pulmonary edema can have various causes and can immediately be a life-threatening emergency. However, it can be treated with percutaneous revascularization if it is caused by renal artery stenosis. This case report reinforces the importance of accurate and immediate diagnosis when dealing with flash pulmonary edema. This case emphasizes the potential therapeutic benefit of renal artery stenting in the management of flash pulmonary edema caused by renal artery stenosis in patients with a single-functioning kidney.

## 1. Introduction

Flash pulmonary edema is an immediate, life-threatening emergency disease in which alveolar space flooding can occur within a few minutes. Flash pulmonary edema can be caused by several pathophysiological processes. Obstructive coronary artery disease, high blood pressure, and renal artery stenosis can cause flash pulmonary edema. Renal artery stenosis, particularly bilateral renal artery stenosis, is one of the leading causes of flash pulmonary edema.^[1]^ Herein, we present a case of recurrent flash pulmonary edema caused by unilateral renal artery stenosis and shrinkage of the contralateral kidney due to vascular complications in a patient with Takayasu arteritis.

## 2. Case description

A 78-year-old woman visited the emergency room in February 2021 for sudden onset dyspnea. This was the 5th emergency room visit since 2018 for sudden dyspnea caused by recurrent flash pulmonary edema. There were no remarkable symptoms until the day before the visit to the emergency room; however, the patient’s condition deteriorated abruptly. The underlying comorbidities included heart failure, non-ST-elevation myocardial infarction, chronic kidney disease, hypertension, and diabetes mellitus. Her heart rate and body temperature were 90 beats/minutes and 36.5 °C, respectively. Notably, the blood pressures in both arms were different. The systolic blood pressure of the right and left arms was 180 and 150 mm Hg, respectively, and both diastolic blood pressure measurements were 100 mm Hg. Oxygen saturation (95%) was maintained with a 3-L nasal oxygen supply. Chest computed tomography (CT) revealed pulmonary edema and pneumonia. The serum creatinine level was 1.63 mg/dL, and estimated glomerular filtration rate (eGFR) was 30.3 mL/minutes/1.73 m^2^. Her baseline creatinine level and eGFR were 1.2 mg/dL and approximately 40 mL/minutes/1.73 m^2^, respectively. The pro-B-type natriuretic peptide level exceeded the maximum value (>70,000 pg/mL) that could be measured at our hospital. The pulmonary edema worsened after half a day, and the serum creatinine level increased rapidly to 2.24 mg/dL (the eGFR was 20.6 mL/minutes/1.73 m^2^).

**Figure 1.**
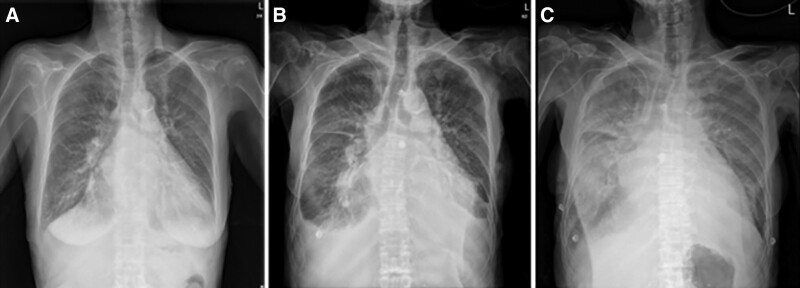
Serial change of chest radiography during the admission period. The chest radiograph demonstrates acute exacerbation of uncontrolled flash pulmonary edema during hospitalization. (A) Day 36 of admission. (B) Day 38 of admission. (C) Day 39 of admission.

She was admitted to the intensive care unit (ICU) of the cardiology department and started on continuous renal replacement therapy (CRRT) to control pulmonary edema. After admission to the ICU, her pulmonary edema improved with CRRT, and she was transferred to the general ward after 5 days of treatment in the ICU. In the general ward, volume control was maintained using diuretics and evaluations were conducted to identify the cause of clinical deterioration. However, approximately 2 weeks after transfer to the general ward, her kidney function deteriorated again with increased diuretic demand, and she experienced rapid worsening of pulmonary edema (Fig. 1). As a result, the patient was readmitted to the ICU 1 month after the initial admission and underwent CRRT again.

Since 2018, she has undergone coronary angiography 3 times during hospitalization for recurrent pulmonary edema. In 2018, a stent was inserted in the proximal right coronary artery, and another was inserted in the proximal left anterior descending coronary artery in 2019. In coronary angiography performed after the recurrence of pulmonary edema in 2020, the already-inserted coronary artery stents were patent, and approximately 60% of the occlusion was confirmed in other coronary arteries; therefore, there was no need for additional coronary artery stent insertion. Transthoracic echocardiography performed in 2020 revealed a left ventricular ejection fraction of 47%, which was not significantly different from before. Following this fifth episode of pulmonary edema, the left ventricular ejection fraction was 43%, which was slightly lower than that in the previous examination; however, no additional signs of coronary artery obstruction were found.

**Figure 2.**
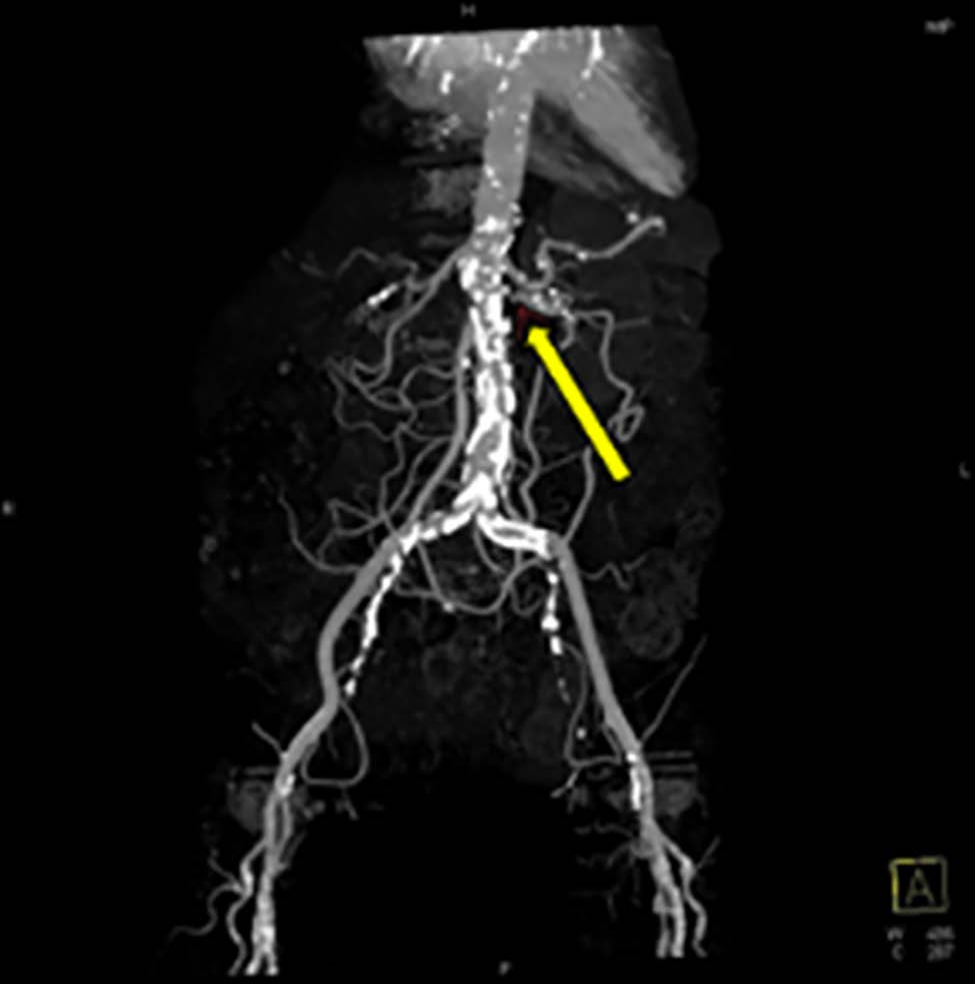
Computed tomography angiography reveals severe stenosis at the left renal artery orifice (yellow arrow).

**Figure 3.**
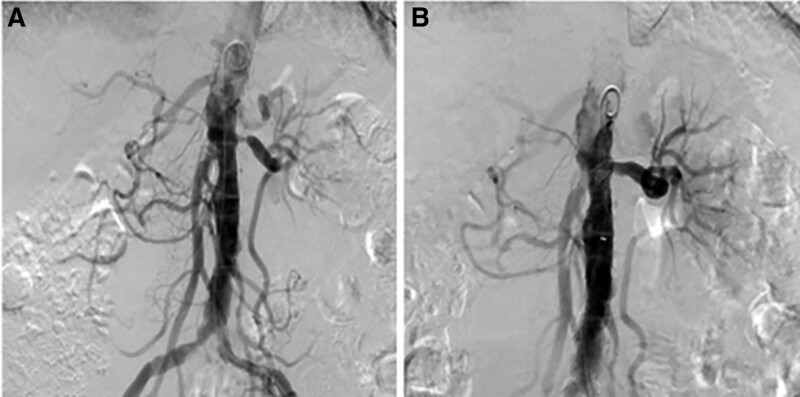
Percutaneous transluminal angioplasty and stenting are performed at the left renal artery stenosis. (A) Selective renal angiography is performed, which reveals stenosis at the orifice of the left renal artery. (B) Angiography also demonstrates successful implantation to treat the stenosis.

The patient was referred to the nephrology department for management of edema and acute kidney injury. To evaluate the cause of the recurrent pulmonary edema, CT renal angiography was performed to assess the renal artery in a patient who had undergone kidney Doppler sonography several months previously, with no findings of a decreased resistive index. On CT, the right kidney was severely atrophic, measuring 6.3 cm, whereas the left kidney appeared relatively normal at 10 cm. Atherosclerosis-induced luminal narrowing and stenosis in the right renal artery and stenosis in the orifice of the left renal artery were observed (Fig. 2). Further history-taking revealed that the patient had recently been diagnosed with Takayasu arteritis. Renal artery luminal narrowing and stenosis may be attributed to Takayasu arteritis. Percutaneous transluminal angioplasty and stenting were performed for the stenosis of the left renal artery (Fig. 3). The patient’s kidney function (serum creatinine level) rapidly improved from 2.32 mg/dL the day before the procedure to 0.92 mg/dL the day after the procedure. On the second day of left renal artery stent insertion, the daily urine volume increased to approximately 3000 mL and dialysis was stopped. Subsequently, the patient’s kidney function remained stable, and she was discharged on the 4th day after stenting of the left renal artery. The patient had been free of flash pulmonary edema for 2 years, and her kidney function remained stable. In November 2022, transthoracic echocardiography revealed slight improvement in heart failure. Before renal artery stenting, the patient’s left ventricular ejection fraction was 43%, whereas it was 46% in this case.

## 3. Discussion

Flash pulmonary edema, which can rapidly cause respiratory failure, can occur in patients with renal artery stenosis. In the absence of normal renal function in response to pressure natriuresis, patients with hemodynamically severe bilateral or solitary renal artery stenosis may exhibit a volume overload.^[2]^ Patients with a solitary kidney may develop pulmonary edema due to angiotensin-mediated vasoconstriction induced by increased left ventricular afterload. Our patient had a single-functioning kidney with an atrophied and nonfunctioning right kidney. It appears that renal artery stenosis develops in such a single-functioning kidney and induces flash pulmonary edema.

Takayasu arteritis is a chronic vasculitis that mostly involves the aorta and its major branches, including the brachiocephalic, carotid, subclavian, vertebral, and renal arteries as well as the coronary and pulmonary arteries. Common complications of this disease include Takayasu retinopathy, secondary hypertension, and aortic or arterial aneurysms.^[3]^ In many cases, renal artery anomalies may be the key cause of newly diagnosed hypertension in many cases.^[4]^ Moreover, Takayasu arteritis causes renal artery stenosis and leads to sudden onset of pulmonary edema.^[5]^

**Figure 4.**
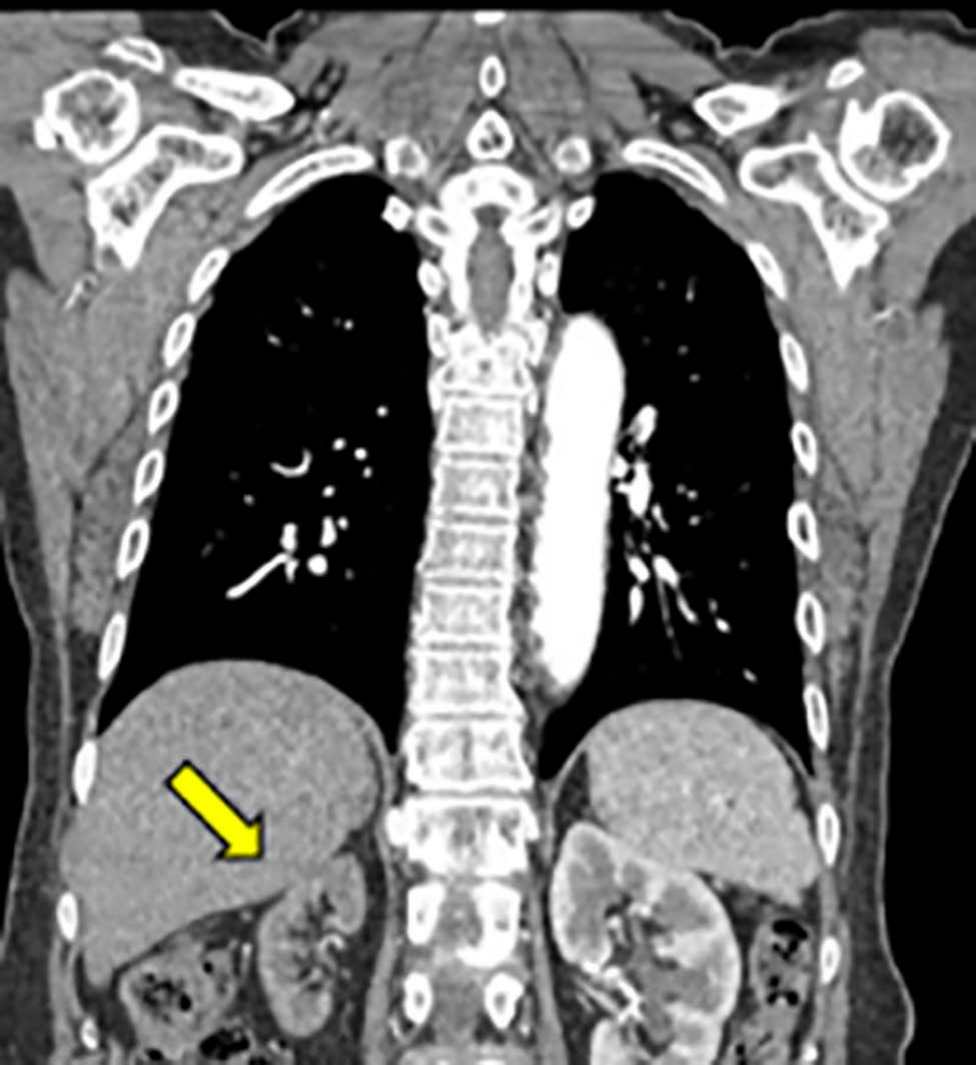
Right kidney atrophy (yellow arrow) was observed on computed tomography performed 2 months prior to the first percutaneous coronary intervention procedure.

**Figure 5.**
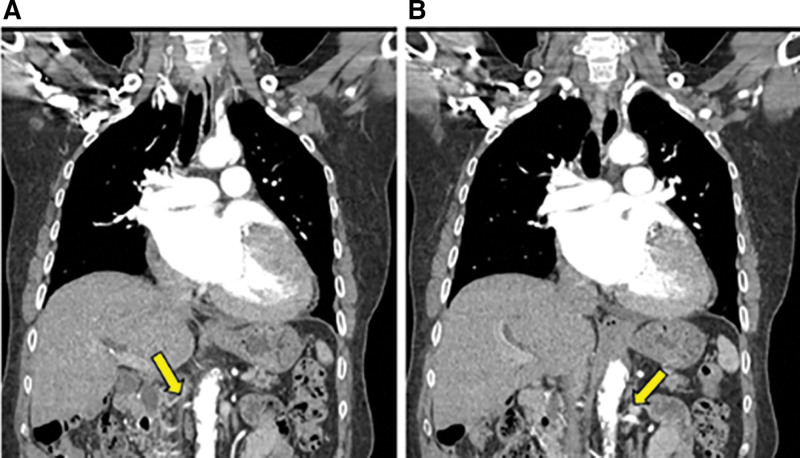
Chest computed tomography before coronary artery stent insertion. The orifice of both renal arteries shows stenosis. (A) Right kidney artery stenosis. (B) Left kidney artery stenosis.

Renal artery stenosis can cause cardiac destabilization syndromes. The most representative example is the flash pulmonary edema observed in this case. The mechanism by which renal artery stenosis leads to cardiac destabilization syndrome is believed to be uncontrolled hypertension and volume retention, which can lead not only to heart failure, but also to acute coronary syndrome.^[6]^ A similar course was observed in this patient. She had experienced repeated intermittent chest pain, dyspnea, and uncontrolled high blood pressure since 2018, and CT angiography of the chest conducted on October 24, 2018, revealed no major abnormalities in the coronary artery; however, upon reviewing the CT chest angiography images, there were suspicious areas of atrophy in the right kidney (Fig. 4) and stenosis in the orifice of both renal arteries (Fig. 5). A few months later, the patient underwent percutaneous coronary artery intervention for the first time because of the same symptoms. Based on the information presented earlier, it is possible that the patient’s repeated visits to the emergency room with flash pulmonary edema and subsequent percutaneous coronary artery intervention were triggered by the renal artery stenosis.

Renal artery stenting may be a potential therapeutic option for the management of congestive heart failure or unstable coronary syndrome, as supported by the mechanisms discussed earlier.^[7]^ According to the Canadian Cardiovascular Society Angina Classification and New York Heart Association Functional Classification, successful renal stent implantation led to a significant drop in blood pressure and management of angina symptoms in 88% of patients with established coronary artery disease and in at least 1 renal artery with hemodynamically significant stenosis. Additionally, when comparing the group that underwent coronary intervention with renal stent insertion with the group that received renal stent insertion alone, no incremental therapeutic benefit was observed.

Management of renal artery stenosis resulting from various causes, including Takayasu arteritis, is largely divided into medical treatment and revascularization. According to the guidelines presented by the American Heart Association/American College of Cardiology Committee for the treatment of renal artery stenosis, medical treatment is the first-line treatment for hypertension and preservation of renal function, whereas percutaneous revascularization is the first-line treatment for hemodynamically significant renal artery stenosis, recurrent unexplained congestive heart failure, and sudden unexplained pulmonary edema.^[8,9]^

There are some limitations in our study. As previously stated, percutaneous revascularization is not always the first-line treatment for renal artery stenosis; therefore, our strategy may not be applicable to all patients. However, revascularization plus medical therapy rather than medical therapy alone should be considered when recurrent flash edema in a single-functioning kidney. In addition, our patient had a history of Takayasu arteritis and recurrent episodes of flash pulmonary edema. It would have been better if the renal artery as well as the coronary arteries of the heart had been evaluated early.

Flash pulmonary edema can have various causes and can immediately be a life-threatening emergency. However, it can be treated with percutaneous revascularization if it is caused by renal artery stenosis. This case report reinforces the importance of accurate and immediate diagnosis when dealing with flash pulmonary edema. Even in patients with preexisting obstructive coronary artery disease, it is necessary to evaluate the possibility of renal artery stenosis when recurrent flash pulmonary edema occurs. Furthermore, it is necessary to consider the possibility that the accompanying coronary artery disease is caused by renal artery stenosis.

## Acknowledgments

This work was supported by National Research Foundation of Korea (NRF) grants funded by the Korean government (MSIT:2021R1F1A1061372) and a clinical research grant from Pusan National University Hospital in 2023.

## Author contributions

**Conceptualization:** Hyo Jin Kim.

**Data curation:** Hyo Jin Kim, Ji Eun Cheon.

**Investigation:** Ji Eun Cheon.

**Supervision:** Hyo Jin Kim.

**Validation:** Hyo Jin Kim, Ji Eun Cheon.

**Writing** – original draft: Ji Eun Cheon.

**Writing** – review & editing: Hyo Jin Kim.
